# STIM1 activates CRAC channels through rotation of the pore helix to open a hydrophobic gate

**DOI:** 10.1038/ncomms14512

**Published:** 2017-02-21

**Authors:** Megumi Yamashita, Priscilla S.-W. Yeung, Christopher E. Ing, Beth A. McNally, Régis Pomès, Murali Prakriya

**Affiliations:** 1 Department of Pharmacology, Feinberg School of Medicine, Northwestern University, Chicago, Illinois 60611, USA; 2 Molecular Structure and Function, Hospital for Sick Children, University of Toronto, Toronto, Ontario, Canada M5G 0A4; 3 Department of Biochemistry, University of Toronto, 101 College Street, Toronto, Ontario, Canada M5G IL7

## Abstract

Store-operated Ca^2+^ release-activated Ca^2+^ (CRAC) channels constitute a major pathway for Ca^2+^ influx and mediate many essential signalling functions in animal cells, yet how they open remains elusive. Here, we investigate the gating mechanism of the human CRAC channel Orai1 by its activator, stromal interacting molecule 1 (STIM1). We find that two rings of pore-lining residues, V102 and F99, work together to form a hydrophobic gate. Mutations of these residues to polar amino acids produce channels with leaky gates that conduct ions in the resting state. STIM1-mediated channel activation occurs through rotation of the pore helix, which displaces the F99 residues away from the pore axis to increase pore hydration, allowing ions to flow through the V102-F99 hydrophobic band. Pore helix rotation by STIM1 also explains the dynamic coupling between CRAC channel gating and ion selectivity. This hydrophobic gating mechanism has implications for CRAC channel function, pharmacology and disease-causing mutations.

## Abstract (web summary)

Store-operated Ca^2+^ channels (CRAC) are involved in several cellular functions. Here the authors identify a hydrophobic gate in the CRAC pore and show that CRAC activation by STIM1 involves rotation of the pore helix that hydrates this region to allow ion passage through the pore.

A central aspect of ion channel function involves its gating mechanism—the process of opening and closing of the pore through conformational motions of the channel gate. Many types of gates have been described in ion channels including rigid body structures that sterically block pores^1^, hydrophobic residues that present energy barriers for solvated ions^2^, and reversible salt bridges across the pore that function as electrostatic switches^3^. The gating of store-operated CRAC channels has attracted considerable interest due to their unusual mode of activation that occurs through the depletion of endoplasmic reticulum (ER) Ca^2+^ stores and their critical importance for human health^4^. CRAC channels generate Ca^2+^ signals that drive a wide range of effector functions including transcription, motility and proliferation^4^. Their clinical relevance is underscored by loss- and gain-of-function mutations that lead to devastating immunodeficiencies, muscle weakness and bleeding disorders^4^,^5^, and a growing list of diseases from asthma to pain for which CRAC channels are being explored for the development of new therapeutics^6^,^7^. Yet, the basic question of how CRAC channels open following store depletion remains poorly understood.

The prototypic CRAC channel is formed by Orai1, a plasma membrane protein with four transmembrane domains. Following store depletion, activation of CRAC channels occurs through interaction of its cytoplasmic N and C termini with the ER Ca^2+^ sensor, STIM1, resulting in both molecules gathering in closely apposed sites at the ER–plasma membrane junctions^4^. At the structural level, the CRAC channel pore is composed of six Orai subunits^8^,^9^,^10^ with the transmembrane 1 (TM1) helices of each subunit forming the Ca^2+^ selective pore and the other transmembrane helices (TM2-4) arranged in concentric layers around the innermost TM1 segments^4^,^10^. The Ca^2+^ selectivity filter resides towards the extracellular end of the pore and is formed by a ring of six glutamate residues (E106 in hOrai1) that bind Ca^2+^ ions^11^,^12^,^13^,^14^,^15^. This overall structure, of a narrow central pore flanked by TM1 helices, agrees well with previous electrophysiological and biochemical studies that have probed the functional pore architecture of Orai1 channels^16^,^17^.

Much less is understood, however, of the identity of the CRAC channel gate and how it opens to permit ion conduction. Some studies have implicated a role for positively charged residues in the inner pore, with channel activation postulated to involve dilation of this pore region^10^,^18^. However, this viewpoint has been challenged^19^ based on observations showing that Orai1 channels with a deletion of the inner pore containing a putative gate still retain a barrier to ion permeation^19^,^20^. Instead, several laboratories have postulated that the CRAC channel gate resides in a hydrophobic segment towards the extracellular side of the pore. In particular, based on the constitutive ion permeation evoked by mutations of V102 (V102C/A/S/T)^21^,^22^, this residue was proposed to function as a hydrophobic gate by presenting a free energy barrier for water and cations^21^,^23^. Consistent with this overall concept, lanthanide resonance energy transfer (LRET) measurements implicate V102 in STIM1-mediated gating^19^ and molecular simulations show that pore hydration is markedly increased in the constitutively permeant V102A mutant^23^. However, how V102 regulates the opening of the pore, and whether this occurs through pore dilation or rearrangement of the non-polar side-chains is unknown.

Here, we use state-dependent accessibility, structure-function analysis, and molecular dynamics simulations (MD) to examine the conformational changes that underlie the opening of the CRAC channel pore. Our results indicate that the CRAC channel gate is formed by two rings of closely spaced hydrophobic pore-lining residues (V102 and F99), which together contribute to a dewetting barrier that prevents ion conduction in closed channels. STIM1 binding leads to a modest rotation of the pore helix, which displaces F99 residues away from the central pore axis to disrupt the V102-F99 hydrophobic band, thereby increasing pore hydration and permitting ion conduction.

## Results

### Experimental concept

**Figure 1:**
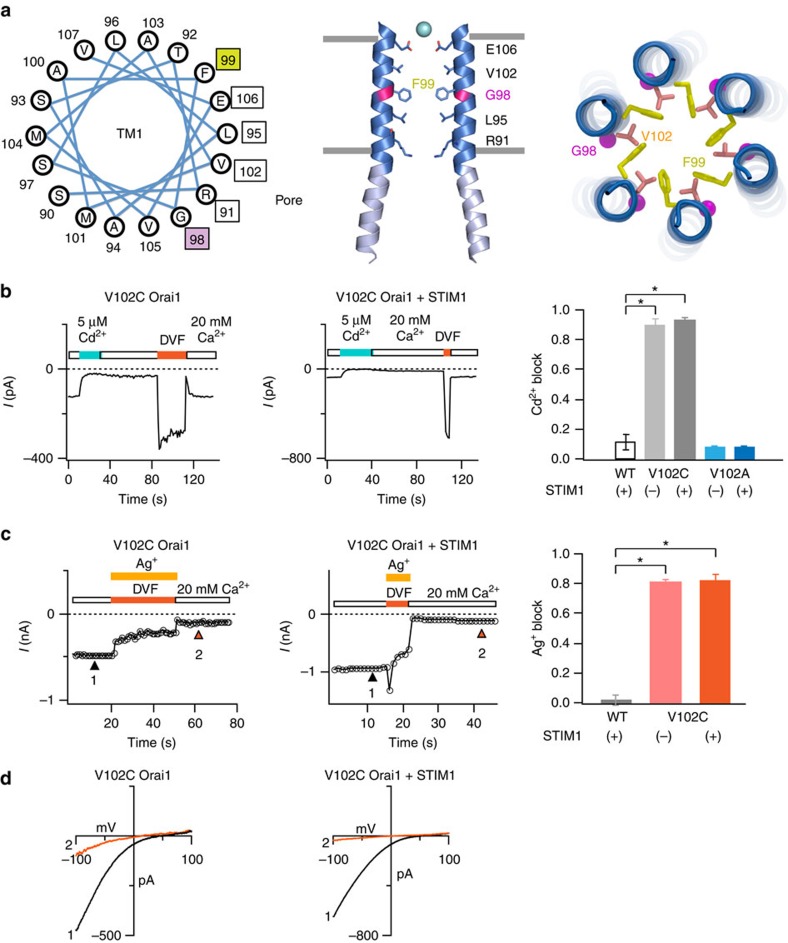
Cd^2+^ and Ag^+^ blockade of V102C Orai1 is not modulated by STIM1. (**a**) A helical wheel representation of residues in the TM1 pore helix (top view). Pore-lining residues previously identified through cysteine scanning in the STIM1-gated Orai1 channel^16^, or in the dOrai crystal structure (PDB code ID 4HKR)^10^, are shown in boxed text. F99, whose corresponding residue in dOrai (F171) was found to be pore-lining in the crystal structure, is shown in green, whereas G98, located 100° away is depicted in pink. The cartoons in the middle panel depict side-view of two TM1 helices showing pore-lining residues. A Ba^2+^ ion is shown in blue above the selectivity filter. The cartoon on the right shows the radial positions of V102, F99 and G98 viewed from the top. (**b**) Cd^2+^ (5 μM) blocks V102C Orai1 current to a similar extent in the absence and presence of STIM1. The indicated Orai1 constructs were expressed in HEK293 cells either with or without STIM1 co-expression. The background block in WT and V102A channels is <10%. *N*=8–16 cells for each condition. *=*P*<0.001 (Student's *t*-test). Values are mean±s.e.m. (**c**) Ag^+^ (130 nM) blocks V102C Orai1 currents to a similar extent in the presence or absence of STIM1. WT Orai1 channels are not affected by Ag^+^ (Supplementary Fig. 1), but the introduction of cysteine at V102 confers sensitivity to Ag^+^ blockade. Blockade by Ag^+^ was measured by comparing current amplitudes in 20 mM [Ca^2+^]_0_ before and following Ag^+^ administration (arrowheads). *N*=4–5 cells for each condition. *=*P*<0.001 (Student's *t*-test). Values are mean±s.e.m. (**d**) Ag^+^ blockade does not alter the current–voltage (*I*–*V*) relationship of Orai1. *I*–*V*s were obtained at the time points indicated by the arrowheads in **c**.

The pore-lining residues in TM1 identified by the *Drosophila* Orai crystal structure^10^ match those found by previous cysteine accessibility analysis^16^ remarkably well with one notable exception. Whereas the dOrai crystal structure (STIM1-free and therefore presumably closed) depicts the bulky hydrophobic residue F171 (equivalent to F99 in hOrai1) to be pore-lining^10^, cysteine accessibility analysis in the STIM1-activated Orai1 channel found that G98 is the pore-lining residue at this locus^16^,^21^. A helical wheel representation of the TM1 residues shows that G98 and F99 are located on opposite sides of the pore helix (Fig. 1a). This difference led us to hypothesize that STIM1 evokes a conformational change that alters the accessibility of these residues, exposing G98 while concealing F99 away from the pore.

To test this hypothesis, we exploited the constitutive activity of V102A/C Orai1 mutants to probe the pore conformational change evoked by STIM1 binding. Previous studies have found that the constitutive activity of V102A/C channels arises from a ‘leaky' gate that eases a hydrophobic free energy barrier for ions in the pore^19^,^21^,^22^. This conclusion is supported by MD simulations, which showed that the V102A mutation increases water and monovalent ion occupancy in the pore^23^, and LRET measurements that imply that the V102A pore is similar in conformation to closed channels^19^. We therefore used the constitutively conducting V102A/C Orai1 mutants as surrogates for probing the resting state of the channel.

To examine changes in the pore conformation caused by STIM1, we engineered cysteine residues into TM1 and examined their accessibility to water-soluble thiol reagents (Cd^2+^ and Ag^+^) applied from the extracellular side of the channel in the presence and absence of STIM1. Persistent blockade of ion conduction by Cd^2+^ requires coordination of this metal ion in the pore by multiple (3 or more) cysteines^21^,^24^,^25^,^26^,^27^. Therefore, the formation of stable Cd^2+^ ‘bridges' provides a rigorous method to assess whether or not cysteines engineered into the TM1 pore helix are oriented towards the pore. Moreover, because the length of each S–Cd^2+^ bond is only ∼2.5 Å^28^,^29^, stable Cd^2+^ blockade indicates that the pore-facing cysteines in question are in close proximity to one another. Ag^+^ is a monovalent cation comparable in size to Na^+^ and K^+^ ions, which permeate readily through CRAC channels in divalent free (DVF) solutions. However, unlike Cd^2+^, Ag^+^ readily reacts with any available free cysteines at water accessible surfaces of proteins, forming single S–Ag covalent bonds. Thus, while modification by Ag^+^ cannot yield information regarding the orientation of the Cys side-chains with respect to one another, a change in channel activity indicates that the residue in question is solvent accessible^30^,^31^. In WT or V102A Orai1 channels, there is negligible blockade with both Cd^2+^ and Ag^+^, (Fig. 1b; Supplementary Fig. 1a). Therefore, this approach provides a relatively clean background in which to study the effects of these reagents in cysteine mutants.

### Accessibility of V102C is unchanged by STIM1

In previous studies, the hydrophobic residue, V102, was implicated as a likely candidate for the channel gate based on the constitutive channel activity evoked by substitutions of V102 to polar residues (V102G/S/T/A/C)^21^. Exactly how V102 might regulate the closed–open transition is, however, puzzling. From the standpoint of an ion in the pore, V102 is located in the middle of the pore-facing TM1 surface (Fig. 1a). On the basis of this disposition, the exposure of V102 to the pore is not likely to be significantly altered by modest conformational changes in TM1. Consistent with this expectation, neither Cd^2+^ nor Ag^+^ blockade in V102C channels was affected by STIM1 (Fig. 1b–d). In particular, the rate constants of Cd^2+^ blockade, which reflect the rate at which Cd^2+^ interacts with its ligands, were unchanged by STIM1 (Supplementary Fig. 2). These results suggest that V102C side-chains are not concealed into the protein core by STIM1 binding and continue to be pore-facing in both closed and open channels. How then could V102 regulate ion conduction? One possibility is that STIM1 binding dilates the pore to diminish the hydrophobic barrier at V102. But this scenario seems unlikely given the short length of S–Cd^2+^ bonds^28^,^29^ and in light of previous findings showing that STIM1 binding actually reduces the minimal pore diameter of V102C Orai1 channels^21^. An alternate possibility is that V102, at least by itself, does not comprise the channel gate. In this scenario, the barrier that dynamically regulates ion permeation must reside elsewhere in the ion conduction pathway.

### STIM1 differentially affects Cd^2+^ accessibility of F99C and G98C

**Figure 2:**
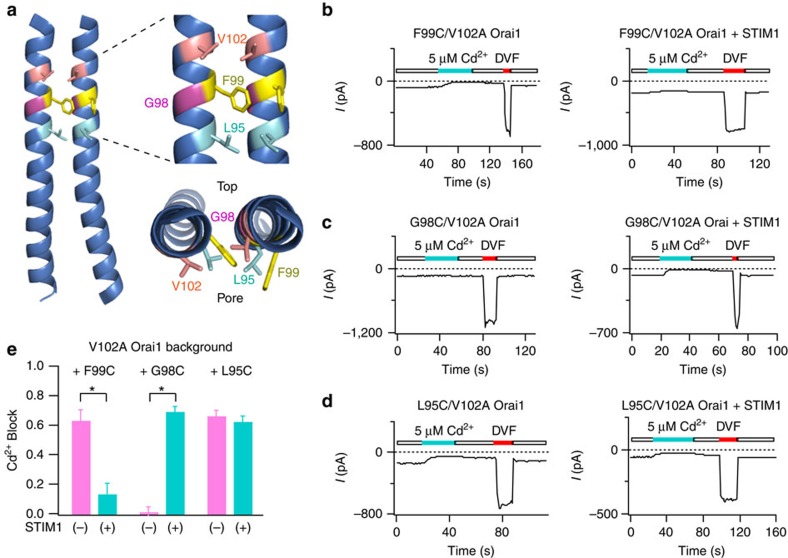
STIM1 binding alters the accessibility of G98C and F99C in opposite ways. (**a**) Cartoons showing side- and top-views of two neighbouring Orai1 subunits based on the crystal structure of *Drosophila* Orai. The predicted positions and relative orientations of V102, G98 and F99 are expanded on the right for clarity. (**b**) Cd^2+^ (5 μM) strongly blocks F99C/V102A currents in the absence, but not presence of STIM1. (**c**) By contrast, Cd^2+^ potently blocks G98C/V102A currents in the presence, but not absence of STIM1. (**d**) Cd^2+^ blocks L95C/V102A Orai1 currents to similar extent in the presence and absence of STIM1. (**e**) Summary of Cd^2+^ blockade in F99C, G98C and L95C mutants. In each case, block was quantified by measuring the current in 20 mM [Ca^2+^]_0_ Ringer's solution before and at the end of Cd^2+^ administration. Each trace shows a plot of the Orai1 currents at −100 mV measured during pulses delivered every second. *: *P*<0.001. (Student's *t*-test). *N*=4–9 cells. Values are mean±s.e.m.

To distinguish between these possibilities, we turned to the other pore-lining residues in the hydrophobic patch, notably F99, L95 and the nearby G98 (Fig. 2a). For these studies, we used the constitutive activity of the V102A mutant to study the accessibility of F99C, G98C and L95 in the presence and absence of STIM1. Consistent with results previously seen in the F99C single mutant activated by STIM1 (ref. 16), we found that Cd^2+^ (5 μM) administered to cells co-expressing F99C/V102A Orai1 and STIM1 evoked very little blockade (Fig. 2b). Strikingly, however, the same concentration of Cd^2+^ evoked large inhibition of F99C/V102A currents in the absence of STIM1 (Fig. 2b,e). Cd^2+^ inhibition was poorly reversible and required a wash with an EDTA-containing divalent-free solution (DVF) to chelate the metal ion (Fig. 2b) indicative of stable Cd^2+^ coordination in the pore. Thus, in the absence of STIM1, F99C side-chains can readily coordinate Cd^2+^ ions flowing in the pore to block permeation, but the addition of STIM1 diminishes Cd^2+^ binding.

In G98C/V102A Orai1 channels, however, Cd^2+^ blockade showed precisely the opposite pattern. Here, Cd^2+^ did not significantly inhibit G98C currents when the mutant was expressed alone, but the addition of STIM1 greatly increased its sensitivity to Cd^2+^ block (Fig. 2c,e). As with F99C/V102A, blockade by Cd^2+^ required a wash with a DVF solution to fully reverse the inhibition. These results indicate that the G98C side-chains are pore-facing in the presence of STIM1, but not in its absence. Thus, STIM1 binding alters the accessibility of G98C and F99C to Cd^2+^ in opposite ways, a finding that is not congruent with simple pore dilation.

L95 is the deepest of the three hydrophobic pore-lining residues in the pore. Like V102C, L95C also did not show changes in Cd^2+^ accessibility by STIM1 (Fig. 2d,e; Supplementary Fig. 2). Collectively, these results show that STIM1 binding profoundly alters Cd^2+^ binding to the peripherally positioned F99C and G98C residues, but not to the more centrally positioned V102C or L95C residues. Further, the change in the pattern of Cd^2+^ blockade in G98C and F99C suggests that F99C side-chains reorient away from the pore, whereas those of G98C move into the pore to better coordinate Cd^2+^ binding. The most plausible conformation change consistent with this pattern is a modest rotation (∼20°) of the TM1 helix in the counter-clockwise direction as viewed from the top (see ‘Discussion' section).

Is the magnitude of this conformational change large enough to completely conceal F99C into the protein core during STIM1 gating? We addressed this question by examining the accessibility of F99C to Ag^+^. These experiments showed that Ag^+^ blocks F99C/V102A currents both in the presence and absence of STIM1 (Supplementary Fig. 1b). Thus, F99C is solvent accessible in both states. Ag^+^ also blocked G98C/V102A currents in the presence of STIM1. However, in the absence of STIM1, Ag^+^ paradoxically potentiated G98C Orai1 currents (Supplementary Fig. 1c). Although the details of how this occurs are not known, a simple interpretation is that G98C is also accessible to Ag^+^ in the ‘resting' state of the channel, but because of its side-facing orientation away from the pore, polar modifications of G98 enhance rather than block permeation. Notably, this effect is qualitatively analogous to the constitutive channel activity observed by introducing polar amino acids at this position (discussed below, Fig. 6). Taken together, these results indicate that the side-chains of G98C and F99C are not buried into the protein core but remain exposed to solvent both in the presence and absence of STIM1. When considered together with the Cd^2+^ results, these findings indicate that the alteration in the pore conformation induced by STIM1 (rotation of the pore helices) is sufficiently modest that it does not substantively alter solvent exposure of the F99C side-chains, but large enough to eliminate their ability to coordinate Cd^2+^ ions in the pore.

### STIM1 dose-dependence of Cd^2+^ binding to F99C

Previous studies have shown that the activation of Orai channels is highly sensitive to the number of STIMs bound to the channel, with maximal channel activation occurring at a STIM1:Orai1 ratio of ∼2:1 (refs 32, 33). How does this compare to the STIM1-driven pore conformational change at F99C? To explore this question, we measured Cd^2+^ blockade in constructs in which F99C Orai1 channels were tethered either to zero, one, or two functional SOAR (STIM1-Orai1 activating region) domains of STIM1 (ref. 34) (S or SS) as previously described^21^,^33^. Single cysteine mutations of several pore-residues including G98, L95 and R91 did not yield constitutively active currents^21^. However, current measurements in the single F99C mutant were made feasible by the constitutive activity of this mutant (described below; Fig. 4d). In this mutant, Cd^2+^ blockade dose-dependently declined with increasing STIM1 at the channel, with F99C-SS channels, and F99C channels co-expressed with STIM1, displaying weaker Cd^2+^ blockade than F99C-S channels (Supplementary Fig. 3). In turn, F99C-S channels were less sensitive to Cd^2+^ blockade than STIM1-free F99C channels. Thus, both Cd^2+^ blockade and channel activation are co-regulated in a similar manner by STIM1, indicating that the pore-facing orientation of the F99C side-chains is linked to the extent of channel activation by STIM1.

### A Cd^2+^ bridge between G98 and F99 evokes channel activation

**Figure 3:**
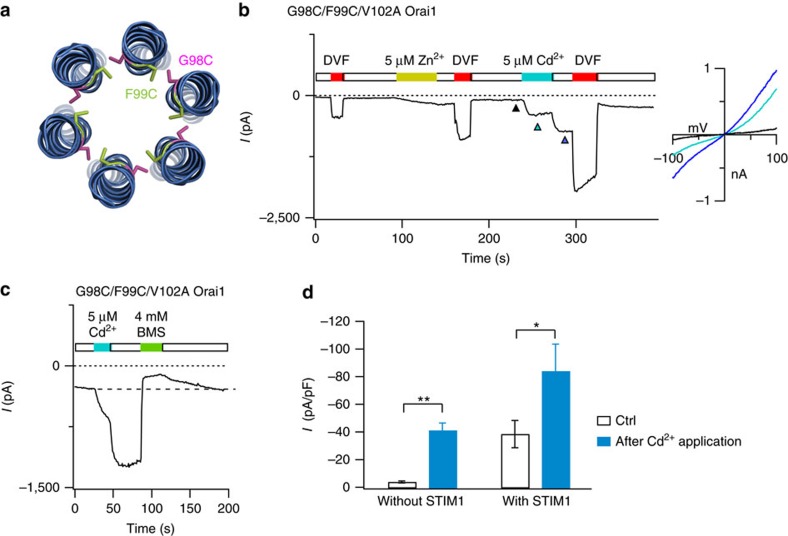
Cd^2+^ and Zn^2+^ activate G98C/F99C channels through a lock-open effect. (**a**) Cartoon representation of the relative positions of engineered cysteine residues at G98 and F99 in neighbouring subunits based on the crystal structure of *Drosophila* Orai. The native residues were altered using the mutagenesis tool in PyMOL. (**b**) The transition metals ions, Zn^2+^ and Cd^2+^, strongly enhance currents in G98C/F99C/V102A channels. Potentiation caused by Zn^2+^ and Cd^2+^ is partially reversed by washing the cell with DVF solution, indicating that it is caused by a lock-open effect from the trapping of the metal ions in the channel. Note that washout of Cd^2+^ reveals further current enhancement, indicating that Cd^2+^ also blocks the potentiated G98C/F99C double cysteine channels probably by binding to a second, low-affinity site. The *I*–*V* relationships at the time points indicated by the arrowheads are shown in the right graph. (**c**) Potentiation of G98C/F99C/V102A current by Cd^2+^ is rapidly reversed by the reducing agent BMS, likely through destabilization of the thiolate groups trapping Cd^2+^. (**d**) Summary of Cd^2+^ potentiation in G98C/F99C/V102A channels in the absence and presence of STIM1. Cd^2+^ potentiates G98C/F99C/V102A channels to a greater extent in the absence of STIM1 than its presence, likely because F99C side-chains have already moved away from the pore axis in the presence of STIM1*. n*=6 cells per condition. **: *P*<0.001. *: *P*<0.05. Paired *t*-test. Values are mean±s.e.m.

If F99 moves away from the pore axis during STIM1-driven channel activation as suggested by the above results, can the converse process (pore opening) be ‘induced' by displacing the F99 side-chains in the absence of STIM1? Structural models place G98 and F99 of neighbouring subunits in close proximity to each other (Fig. 1a). We exploited this feature to examine whether forcing an interaction between engineered cysteines at these positions could affect ion conduction by displacing F99C side-chains away from the pore. To test this, we applied Cd^2+^ on a G98C/F99C/V102A double cysteine mutant to induce a Cd^2+^ bridge between the G98C and F99C. Remarkably, application of Cd^2+^ in the G98C/F99C double cysteine mutant dramatically enhanced Orai1 current (Fig. 3b,c). Cd^2+^-mediated potentiation in this mutant was persistent, and could only be terminated by a reducing agent, bis(2-mercaptoethylsulfone) (BMS), or slowly reversed by washing cells with a EDTA-containing DVF solution. Similar potentiation of G98C/F99C channels also occurred with the thiol reactive metal ion, Zn^2+^ (Fig. 3b). These results are consistent with a ‘lock-open' effect in which the trapping of Cd^2+^ and Zn^2+^ ions in the channel enhances its activity. Cd^2+^ did not potentiate the G98C or F99C single mutants (Fig. 2b,c), indicating that cysteines at both positions (G98 and F99) are required for Cd^2+^-mediated potentiation. Because Cd^2+^ can only bind multiple, but not single cysteine residues with high affinity^26^,^35^, the marked enhancement of G98C/F99C current suggests that a high affinity metal bridge is formed between these two cysteine residues in neighbouring subunits, causing the F99C side-chains to move away from the pore axis and enhance channel activity.

### Lowering hydrophobicity at F99 promotes ion conduction

**Figure 4:**
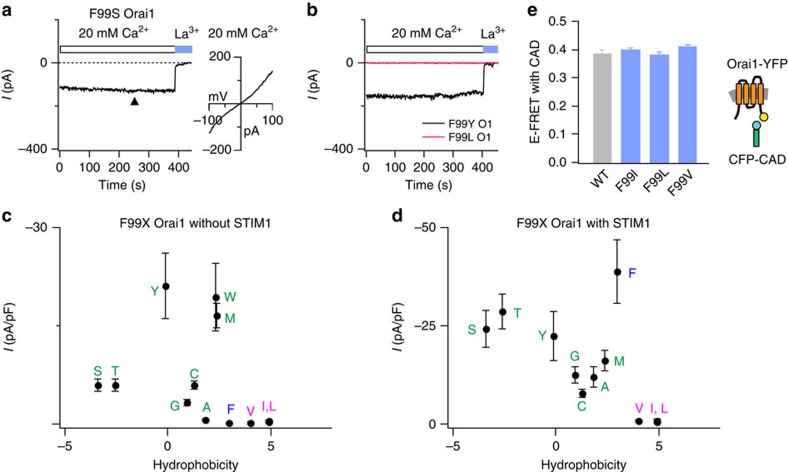
Mutations at F99 produce constitutively permeant Orai1 channels. (**a**,**b**) Time-course of Orai1 current in cells expressing the F99S, F99Y or F99L mutants after whole-cell break-in. Time zero represents the moment of whole-cell break-in. Orai1 mutants were expressed in HEK293 cells without STIM1. The current–voltage relationship of F99S Orai1 at the time point indicated by the arrowhead is shown in the right graph of **a**. (**c**,**d**) Mutational analysis of F99. The current densities measured at steady-state following whole-cell break-in (300–500 s) are plotted against the solvation energies^38^ (kcal mol^−1^) of the substituted amino acid as a measure of their hydrophobicity in the absence (**c**) or presence (**d**) of STIM1 co-expression. Amino acid hydrophobicity increases from left to right. Values are mean±s.e.m. The native residue (Phe) is shown in blue, the constitutively conducting mutants in green, and the non-conducting mutants in magenta. *N*=4–6 cells for each mutant. (**e**) FRET between the indicated Orai1-YFP constructs and CFP–CAD, showing that the mutations do not affect coupling of Orai1 to STIM1. No significant difference was seen in the different mutants compared with WT Orai1. *N*=32–36 cells for each mutant. Values are mean±s.e.m.

The finding that a bulky inline hydrophobic residue, F99, rotates away from the pore axis following STIM1 binding led us to next consider whether this residue is a component of the CRAC channel gate. Consistent with such a role, mutation of F99 to polar or less hydrophobic residues, including Cys, Ser, Thr, Gly, Met and Tyr produced constitutively active Orai1 channels (Fig. 4a–c). Cells expressing these mutants without STIM1 showed standing CRAC currents immediately following whole-cell break-in (Fig. 4a,b; Supplementary Table 1), analogous to the phenotype of the V102C mutant channels previously described^21^. These constitutively permeant F99X channels did not show calcium-dependent inactivation (CDI) (Supplementary Fig. 4) in accordance with the well-established requirement of STIM1 for CDI^36^ and its loss in constitutively conducting Orai1 mutants in the absence of STIM1 (ref. 21). Because Cys, Ser, Thr, Gly, Met, and Tyr are more polar than the native Phe, we inferred that the mutations permit ion conduction by lowering a free energy barrier for the occupancy of water and ions in the pore. By contrast, channels with substitutions to more strongly hydrophobic residues, including Leu, Ile and Val remained closed (Fig. 4b,c; Supplementary Table 1). Of note, substitutions to several other polar and charged residues, including Asp, Glu, Lys, Arg, Gln and Pro resulted in channels that did not traffic to the plasma membrane, effectively precluding analysis of these mutants (Supplementary Fig. 5a). Nevertheless, the functional pattern of the mutants that did express in the plasma membrane strongly suggests that F99 stabilizes the non-conducting (closed) channel state by presenting a hydrophobic barrier in the pore.

### Raising hydrophobicity at F99 impairs pore opening

As noted above, Orai1 channels with F99 substitutions to hydrophobic amino acids (Ala, Val, Ile and Leu) are not constitutively permeant. To examine how STIM1 modifies the activity of these as well as the constitutively conducting F99X mutants, we co-expressed them individually with STIM1 and examined their gating following passive depletion of ER Ca^2+^ stores. In the presence of STIM1, the constitutively permeant F99X mutants showed increases in current beyond their initial amplitude following whole-cell break-in with BAPTA (Supplementary Fig. 6a,b), as expected from the ability of store depletion to further gate these leaky channels. The F99A variant, which is only minimally active in the absence of STIM1, was also activated in a manner similar to WT channels. To our surprise, however, the non-conducting Ile, Leu and Val mutants remained closed even with STIM1 (Fig. 4d). This was not due to a defect in STIM1 binding because FRET tests revealed no difference in the interaction of these mutants with CFP tagged CAD (CRAC activation domain of STIM1 (ref. 37)) (Fig. 4e), or in their ability to recruit CFP–CAD efficiently to the plasma membrane (Supplementary Fig. 5b). Thus, the gating defect in the F99I/L/V mutants is downstream of STIM1 binding.

Ile, Leu and Val are significantly more hydrophobic than the native Phe at position 99 (octanol-water transfer free energies of 4.92 kcal mol^−1^, 4.92 kcal mol^−1^, 4.04 kcal mol^−1^ and 2.98 kcal mol^−1^, respectively^38^), suggesting that the degree of hydrophobicity at this position is likely crucial for preserving store-operated gating. Enhancing the hydrophobicity of the pore in this section evidently produces an energy barrier for ion conduction that is insurmountable, effectively nullifying the ability of STIM1 to ‘open' the pore. Consistent with this interpretation, introduction of an Ala mutation at the nearby V102 position (for example, V102A/F99I), which would be expected to lower the hydrophobicity of this section restored ion permeation in the F99I, F99L and F99V mutants (Supplementary Fig. 7). By contrast, an Ile substitution at V102, expected to preserve the hydrophobic barrier, resulted in non-conducting channels (Supplementary Fig. 7c). Critically, there was no correlation between channel activity and size of the amino acids engineered at F99, indicating that F99 regulates ion conduction through an energetic influence, rather than via steric blockade (Supplementary Fig. 6c,d).

Taken together, these results indicate that the hydrophobicity at position 99 is finely tuned to maintain proper channel function. Decreasing hydrophobicity at this locus leads to constitutively permeant channels, whereas increasing hydrophobicity abrogates STIM1 activation, presumably by increasing the energy barrier for ion conduction imposed by the V102-F99 hydrophobic band. Notably, this phenotype at F99 clearly diverges from the behaviour at the adjacent V102, where substitutions that maintain hydrophobicity of the native Val to other equally strong hydrophobic residues (Ile, Leu and Phe) maintain STIM1 activation^21^.

### Molecular simulations support a gating role for F99

**Figure 5:**
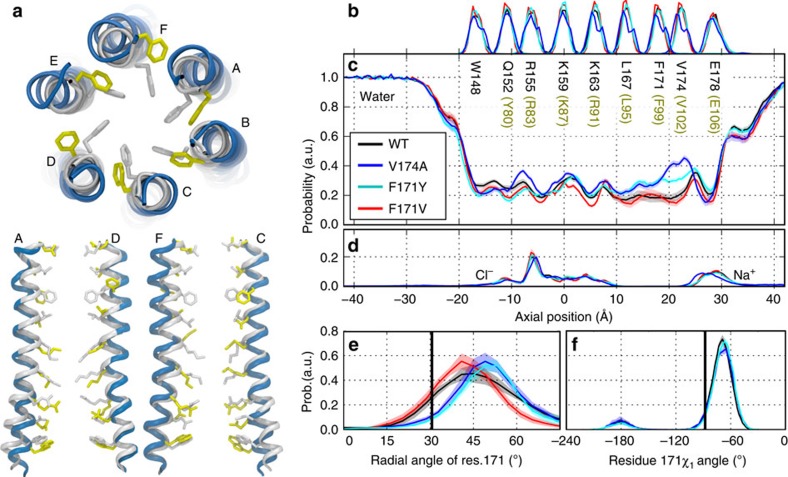
Structural fluctuations and hydration of the dOrai channel pore from MD simulations. (**a**) Superposition of the dOrai crystal structure (white) and a snapshot from MD simulations of WT dOrai highlighting lateral displacement of F171 side-chains (pore helices in blue, F171 in yellow) as viewed from the top. The lower cartoons depict two pairs of diagonal subunits (labelled A, D and F, C) from this snapshot viewed from the side orientation. (**b**) Average distributions of the axial position of Cα atoms for all pore-lining residues. The residues corresponding to human Orai1 are shown in brown. Data in **b**–**f** were computed from simulations of WT (black) and of V174A (blue), F171V (red), and F171Y (aqua) mutant channels. Average distribution of (**c**) water oxygen atoms and (**d**) Na^+^ and Cl^−^ ions along the pore axis. The water occupancies of V174A and F171Y mutants deviate from those of WT and F171V dOrai most significantly in the hydrophobic stretch of the pore. (**e**) Average distribution of the radial angle of residue 171 defined as the angle between the pore axis, the centre of mass of the two helical turns centred at residue 171, and the Cα atom of residue 171. The mean and standard error of mean of these distributions over all simulation repeats in degrees is 46±2 for WT; 43±2 for F171V; 50±2 for V174A; 50±1 for F171Y. The radial angle in the crystallographic structure (31°) is shown as a black vertical bar for reference. (**f**) Average distribution of side-chain torsion χ_1_ of residue 171 in WT, V174A and F171Y mutants. The χ_1_ of residue 171 in the crystallographic structure (−88°) is shown as a black vertical bar for reference. F171V is not included in this analysis as motion involving rotation of the valine side chain χ_1_ angle cannot displace the side chain away from the pore. (**b**–**e**) The traces depict values±s.e.m.

The experimental results showing that hydrophobicity of F99 is critical for controlling ion conduction in Orai1 led us to hypothesize that mutations that decrease hydrophobicity increase water occupancy in the pore, thereby lowering the energy barrier to ion permeation. To test this hypothesis, we performed MD simulations using models constructed from the crystal structure of the *Drosophila* Orai channel (PDB: 4HKR). We performed multiple simulation repeats of the WT, V174A, F171V and F171Y mutants (*Drosophila* Orai residue numbering, corresponding human mutations are V102A, F99V and F99Y, respectively). These simulations revealed several interesting features. First, frequent spontaneous conformational fluctuations involving rotation of the pore helix were observed, resulting in moderate lateral displacements of the F171 side-chains (Fig. 5a). The average helix rotation at residue F171 was shifted by 15±2 degrees in a counter-clockwise direction in the WT channel relative to the crystallographic structure of dOrai and by 12±2 degrees in the F171V mutant. Interestingly, larger counter-clockwise shifts of 19±2 and 20±1 degrees in the average rotation were observed in the constitutively open channels V174A and F171Y, respectively (Fig. 5e). These results reveal spontaneous motions involving pore helix rotation that are enhanced in the constitutively conducting variants. Second, the rotamer populations of the residue 171 χ_1_ angle are comparable for WT, V174A and F171Y models (Fig. 5f). This observation suggests that increased pore helix rotation, rather than enhanced side-chain fluctuations, is responsible for the larger lateral displacements of F171 (or Y171) in the constitutively conducting variants.

Third, the degree to which these conformational fluctuations alter hydration of the pore is reflected in the average distribution of water O atoms along the pore axis (Fig. 5c,d). The WT pore was found to contain water molecules along its entire length, with reductions in water occupancy reflecting the presence of Na^+^ and Cl^−^ binding sites at the selectivity filter and in the basic region, corresponding to residues E178 and R155-K163, respectively (Fig. 5c). The V174A mutation resulted in a large increase in hydration of the hydrophobic zone (L167-V174), supporting the results found in previous MD simulations^23^. A smaller but still significant increase in hydration was observed for the F171Y mutation, whereas the more hydrophobic F171V mutation modestly decreased occupancy of water in the hydrophobic region. Thus, F171V and F171Y induce opposite effects on water occupancy relative to WT channels, consistent with the experimental findings showing that these mutations cause gain or loss of ion permeation. The substantively stronger effect of V174A relative to F171Y on pore hydration correlates well with experimental results indicating that constitutively permeant V102A Orai1 currents are ∼2-fold larger than F99Y currents (∼45 pA/pF in V102A Orai1 (ref. 21) versus ∼22 pA/pF in F99Y Orai1), suggesting that the degree of pore hydration is a key determinant of constitutive ion conduction. Collectively, the simulation results are consistent with the hypothesis that a reduction of hydrophobicity at F99 leads to constitutively permeant Orai1 channels.

### STIM1 binding regulates the Ca^2+^ selectivity of F99 mutants

**Figure 6:**
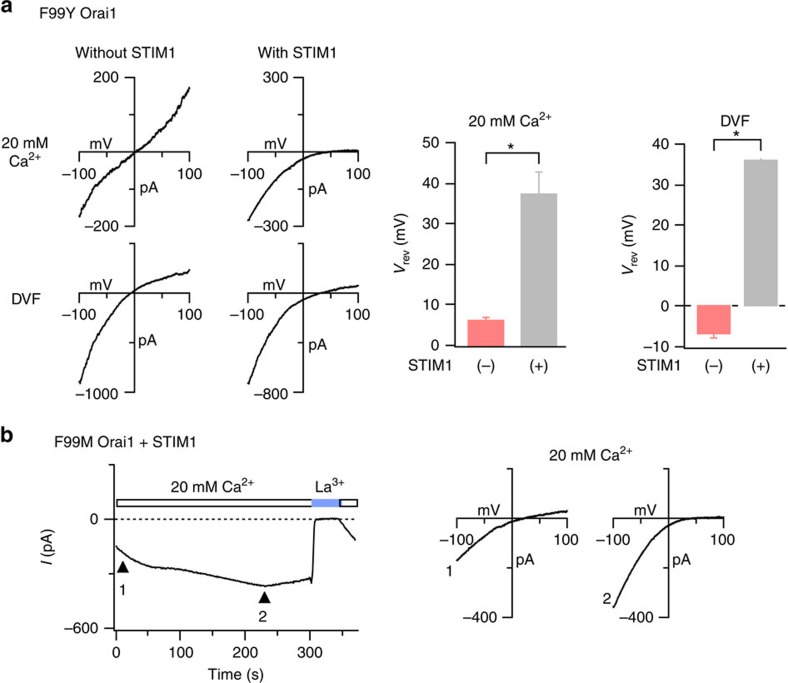
STIM1 regulates the ion selectivity of the constitutively active F99 mutants. (**a**) Current–voltage (*I*–*V*) relationships of F99Y Orai1 currents in 20 mM [Ca^2+^]_0_ and in DVF Ringer's solutions. The bar graphs summarize *V*_rev_ in the presence or absence of STIM1. This change in *V*_rev_ is consistent with increase in Ca^2+^ selectivity and decrease in Cs^+^ permeability^21^. *N*=5 cells for each condition. *=*P*<0.001 (Student's *t*-test). Values are mean±s.e.m. (**b**) Time-dependent change in the ion selectivity of the F99M mutant following whole-cell break-in. Intracellular stores were depleted by dialyzing the cell with 8 mM BAPTA. The left graph plots the time-course of the development of *I*_CRAC_ at -100 mV and the right graphs show the *I*–*V* plots at the times indicated by the arrowheads. Time zero represents the moment of whole-cell break-in.

Ion channels are typically defined by two fundamental properties, their permeation/selectivity behaviour, which is usually considered an invariant property of open state structure, and gating, which involves altering the equilibrium between open and closed channels without influencing the open state structure. In contradiction to this prevailing dogma, the ion selectivity of CRAC channels is dynamic, with STIM1 markedly boosting Ca^2+^ selectivity and lowering Cs^+^ permeability of the constitutively conducting V102C/A Orai1 channels^21^,^22^,^39^. If this attribute arises from rotation of the pore helix following STIM1 binding, then the leaky pores of the constitutively conducting F99X mutants should also show this behaviour. Indeed, as depicted for the F99Y variant, STIM1 markedly shifted the reversal potential (*V*_rev_) of F99Y Orai1 current in both Ca^2+^ and DVF solutions (Fig. 6a). Moreover, the development of I_CRAC_ in cells co-expressing F99M and STIM1 during passive depletion of ER Ca^2+^ stores was accompanied by a gradual shift in *V*_rev_, presumably as interaction of the mutant channels with STIM1 altered their ion selectivity (Fig. 6b). These results indicate that STIM1 binding modifies the ion selectivity of the constitutively permeant F99X channels, probably by modifying the configuration of the Glu selectivity filter through rotation of the pore helix.

### The human mutation G98S causes constitutive Orai1 activation

**Figure 7:**
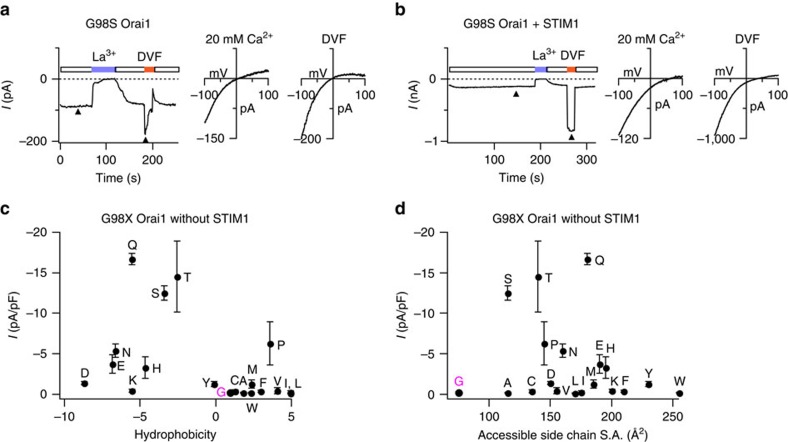
Polar mutations at G98 cause constitutive Orai1 channel activation. (**a**) Constitutively active G98S Orai1 current seen following whole-cell break-in. The arrowheads indicate the time points at which the *I*–*V* relationships of the current are shown in the right graphs. The *I*–*V* plots reveal a non-selective cation current permeable to internal Cs^+^. G98S Orai1 was expressed in HE293 cells without STIM1. (**b**) Co-expression of STIM1 alters the ion selectivity of G98S Orai1 channels. The *I*–*V* plots in 20 mM [Ca^2+^]_0_ and DVF Ringer's solutions (right) show that *V*_rev_ is shifted towards positive potentials. (**c**) Mutations at G98 cause STIM-independent constitutive Orai1 activation. The current densities of the indicated G98X mutant are plotted against the solvation energies^38^ (kcal mol^−1^) of the introduced amino acids as a measure of their hydrophobicity in the absence of STIM1. Amino acid hydrophobicity increases from left to right. Values are mean±s.e.m. (**d**) The constitutive activity of G98X mutants plotted against the accessible side-chain surface area. The native Gly residue is depicted in magenta in **c** and **d**. *n*=3–5 cells for each mutant. Values are mean±s.e.m.

A recent report has described a new gain-of-function human mutation in Orai1, G98S, linked to tubular aggregate myopathy and hypocalcemia in affected individuals of two Japanese families^40^. Consistent with this report, we found that expression of G98S alone in HEK293 cells produced a large constitutively active non-selective Orai1 current (*V*_rev_∼0 mV) (Fig. 7a). Co-expression of STIM1 induced a rightward shift in the *V*_rev_ of G98S Orai1 currents, indicating that STIM1 enhances the Ca^2+^ selectivity and reduces Cs^+^ permeability of this mutant (Fig. 7b), reminiscent of the ‘leaky' gate phenotypes of V102X and F99X mutants in the absence of STIM1.

To examine whether constitutive permeation in the G98S mutant also arises from alterations in pore hydrophobicity, we generated G98X mutants in which the native Gly was substituted with various polar and hydrophobic amino acids. Analogous to the V102X and F99X mutations, the introduction of polar residues (Glu, Asp, Pro, Ser, Thr, Asn and Glu) at G98 resulted in constitutively conducting Orai1 channels (Fig. 7c; Supplementary Table 1). By contrast, introduction of hydrophobic residues (Val, Ile, Leu, Phe, Ala, Trp and even Cys) did not evoke constitutively permeant channels. There was little dependence of the constitutive open phenotype on the side-chain size (Fig. 7d) implying that the conducting phenotype does not arise from steric clash of the exogenous side-chain with the neighbouring TM1 subunits. Taken together, these results indicate that the G98S mutation causes constitutive ion permeation most probably by enhancing hydration of the pore.

## Discussion

CRAC channels are ubiquitously expressed and mediate a diverse array of Ca^2+^-dependent cellular effector functions, yet how they open in response to store depletion is not well-understood. In this study, we investigated the mechanism of the CRAC channel gate and find that V102, and the previously unidentified aromatic residue, F99, function synergistically as a hydrophobic gate to regulate ion conduction through the Orai1 channel pore. Ions entering the channel incur a desolvation penalty in the hydrophobic zone, starting with V102 in the outer pore and continuing with the bulky F99 deeper into the pore^23^. In the closed channel state, this cumulative free energy barrier is strong enough to prevent ion conduction. When STIM1 binds to Orai1, it induces a counter-clockwise rotation of the pore helix, which reorients F99 away from the central pore axis. The smallest extent of TM1 helix rotation required to conceal the Cα atom of F99 away from the pore while revealing G98, located on the opposite face of TM1, without simultaneously affecting the pore exposure of the other TM1 residues is ∼20° (estimate based on the crystallographic structure). Although the pore exposure of the centrally positioned V102 residue would not be appreciably altered by such a moderate rotation, the tangential location of F99 would cause the bulky F99 side-chains to move away from the pore axis. This motion would diminish the free energy barrier for water and ions, thereby permitting ion conduction.

Consistent with this model, MD simulations indicate that a significant amount of pore helix rotation occurs spontaneously with thermal motions even in the absence of STIM1, both in the WT channel and in the three variants considered (Fig. 5e). This finding has several implications. First, it suggests that helix rotation corresponds to a low-manifold protein conformational change well-suited for relaying an allosteric gating mechanism. Second, it is suggestive of a gating process involving conformational selection within an ensemble of pore structures differing in the orientation and degree of exposure of the F99 side chain, rather than a well-defined transition between two rigid conformational states. Third, the results also reveal coupling between the hydration of the hydrophobic stretch of the pore and the lateral displacement of F99. Specifically, the hydrophobic side-chains of F99 and F99V (human equivalent of the drosophila F171V mutant) tend to point towards the pore axis in the less-hydrated, closed state of the pore, whereas enhanced hydration in the V102A and F99Y mutants is concomitant with moderate lateral displacement of the F99 and F99Y side-chains away from the pore axis (Fig. 5c,e). These results should not be taken to imply that the hydrated state of the F99Y and V102A mutants is identical to the open-gate conformation of the channel induced by STIM1. Rather, these mutations appear to induce a shift in the direction of the open state.

We do not know the structural or energetic underpinnings of how STIM1-Orai1 binding induces rotation of the TM1 pore helix, but several explanations are possible. One straightforward possibility is that STIM1 binding to the non-pore-facing surface of the nearby N terminus (residues 73–85)^37^ induces rotation of the TM1 helix located above it. An alternate possibility is that STIM1 binding to the Orai1 C terminus transmits a signal through the transmembrane domains to reorient the TM1 pore helix. In this scenario, pore helix rotation is driven through interactions between the TM1 helix and the flanking TM2 and/or TM3 helices. In a variation of this idea, a third possibility is that the cytoplasmic Orai N terminus interacts with other parts of the channel protein to reorient the nearby pore helix. In each instance, the presence of water molecules around the pore helices may serve to lower the energetic cost of rotating the helix during channel opening. These possibilities are of course not mutually exclusive and more structural and functional studies are needed to explore the scenarios.

**Figure 8:**
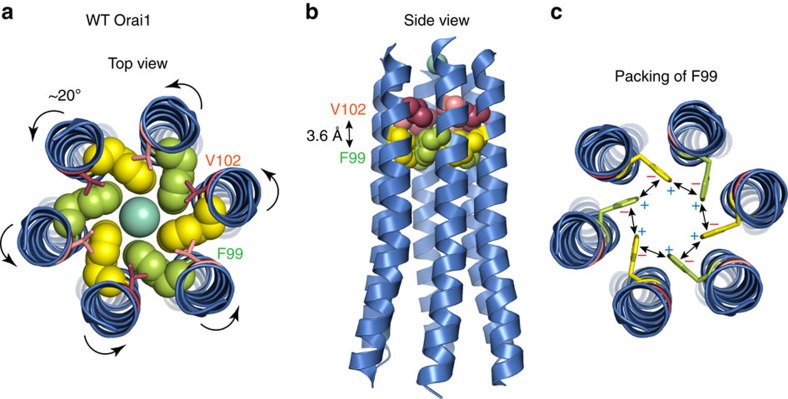
Design features of the hydrophobic gate. (**a**) Top-view of the Orai1 channel pore based on the crystal structure of *Drosophila* Orai showing the predicted positions and orientations of V102 and F99. The side-chain of V102 is depicted as a stick and that of F99 in a space-filling model in this cartoon. Arrows denote the counter-clockwise rotation of the pore helix induced by STIM1. (**b**) Side-view of the Orai1 channel pore. Side-chains of V102 and F99 are shown in a space-filling model in this representation. The distance between the closest atoms of V102 and F99 of neighbouring subunits is ∼3.6 Å. (**c**) Potential edge-face cation–π interactions between F99 residues of neighbouring subunits. Pore helix rotation is expected to destabilize the interactions shown in **b**) and **c**), and release F99 side-chains to move away from the pore axis to open the pore.

Inspection of the crystallographic structure of dOrai shows several noteworthy design features in the construction of the Orai channel gate that could help optimize its function. First, the side-chains of F99 in one subunit appear to engage the hydrophobic V102 side-chains of the neighbouring subunit (Fig. 8a,b). This interaction could, in theory, stabilize the pore-exposed orientation of the F99 side-chains. Second, the side-to-face orientation of neighbouring Phe residues in the closed channel raises the possibility of cation-π interactions between the edge and face of adjacent Phe residues (Fig. 8b)^41^,^42^. These edge-face energetic interactions could stabilize the Phe side-chains towards a pore-facing configuration in closed channels. Third, the 100° separation of F99 and G98 side-chains means that these residues in neighbouring subunits are essentially facing each other, an arrangement that should, in theory, allow the bulky F99 to move around freely in the space created by the compact Gly residue located on the neighbouring subunit during helix rotation (Fig. 1a). Finally, the mutational analysis shows that substituting F99 with any other amino acid impairs channel function, yielding either constitutively permeant or non-conducting channels. Thus, the hydrophobicity at this locus is finely tuned to permit ion conduction in the open configuration while ensuring that the barrier is sufficiently strong to block conduction in closed channels.

The design of the pore described here also helps explain why the G98S human disease-causing Orai1 mutation linked to tubular aggregate myopathy and hypocalcemeia is constitutively permeant^40^. We find that Ser and a variety of hydrophilic (but not hydrophobic) mutations at G98 elicit constitutive ion permeation, analogous to the results seen at V102 and F99. The side-chain dependence of constitutive conduction suggests that the G98X mutations likely exert their effects by enhancing the hydrophilic environment of the pore to produce a leaky gate. Interestingly, previous work has suggested that G98 may function as a gating hinge, and that a possible kink at this position may favour opening of an inner gate^18^,^43^. In our analysis of local bends/kinks in the pore helices of dOrai using MD simulations, we did not observe any evidence of bending/kinking at G170 (Fig. 5a). Hence, we favour the idea that the Gly at this position in each subunit simply serves to provide space to facilitate movement of the opposite-facing Phe residues located on the neighbouring subunit rather than functioning as hinge.

Finally, our results also help explain the mechanistic basis of the dynamic regulation of Orai1 ion selectivity by STIM1 and specifically the enhancement of Ca^2+^ selectivity and reduction in Cs^+^ permeability seen in the constitutively permeant V102X, F99X and G98X mutants. In a previous study examining the leaky V102C mutant channel^21^, we showed that this change is closely correlated to the extent of channel activation by STIM1: both are boosted in a dose-dependent fashion with increasing STIM1, suggesting that the STIM1-driven conformational change that activates the hydrophobic gate also affects the selectivity filter (E106). The finding that STIM1 drives rotation of the pore helix now provides an explanation for this conformational coupling. Counter-clockwise rotation of the pore helix would be expected to alter the orientation of Glu side-chains forming the selectivity filter and therefore change Ca^2+^ selectivity. Furthermore, this finding implies that the counter-clockwise rotation embraces at least three turns of the pore helix (E106 to F99) and may possibly involve an even larger swath of TM1 stretching into the cytoplasmic extension helix. This idea needs further testing.

The hydrophobic gating model that we propose here for CRAC channels is reminiscent of the gating mechanism proposed for the nicotinic acetylcholine receptor (nAChR), where two rings of hydrophobic side-chains at the midpoint of the pore function as the channel gate^2^. As we propose for the CRAC channel, because the gate corresponds to a free energy barrier opposing ion movement, a small rotation (15°) and flexing of the pore helices is sufficient to shift the energetic landscape from a non-conducting to a fully conducting state^44^. Likewise, in the magnesium ion channel CorA, gating involves small-amplitude fluctuations in pore helix tilts resulting in sharp wetting and dewetting transitions of a 12-Å hydrophobic stretch which, in turn, strongly modulate the free energy barrier to Mg^2+^ permeation^45^. Finally, in the mechanosensitive channel MscL, two rings of hydrophobic side-chains, Ile and Val, have been proposed to control ion conduction^46^. On the basis of these examples and the results presented here for CRAC channels, it is possible that the hydrophobic gating mechanism might be a common feature across a wide variety of ion channel families.

## Methods

### Cells

HEK293 cells were maintained in suspension in a medium containing CD293 supplemented with 4 mM GlutaMAX (Invitrogen) at 37 °C, 5% CO_2_. For imaging and electrophysiology, cells were plated and adhered to poly-L-lysine coated coverslips at the time of passage, and grown in a medium containing 44% DMEM (Corning), 44% Ham's F12 (Corning), 10% fetal calf serum (HyClone), 2 mM glutamine, 50 U ml^−1^ penicillin and 50 μg ml^−1^ streptomycin.

### Plasmids and transfections

The F99X and V102C Orai1 mutants employed for electrophysiology were engineered into a pEYFP-N1 vector (Clonetech) to produce C-terminally tagged Orai1-YFP proteins^47^. G98X mutations were engineered into either CFP-Orai1 (cloned into the pReceiver M32 vector; purchased from GeneCopoeia) or Orai1-YFP constructs. No difference in functional properties was noted between the two constructs. mCherry-STIM1 and CFP–CAD were kind gifts of R.S. Lewis (Stanford University, USA). The ORAI1–S–eGFP and ORAI1–SS–eGFP constructs were kind gifts of T. Xu^33^ (Chinese Academy of Sciences, China). Unlabelled STIM1 used for the electrophysiology was obtained from Origene Technologies (Rockville, MD, USA). All mutants were generated by the QuikChange Mutagenesis Kit (Agilent Technologies) and the mutations were confirmed by DNA sequencing. Primer sequences used for generating Orai1 mutants used in this study are listed in Supplementary Tables 2, 3 and 4.

For electrophysiology, the indicated Orai1 constructs were transfected into HEK293 cells either alone (100–200 ng DNA per coverslip) or together with STIM1 (100 ng Orai1 and 500 ng STIM1 cDNA per coverslip) and currents were recorded 24 h later. For FRET and fluorescence imaging experiments, Orai1-YFP (100 ng per coverslip) and CFP–CAD (100 ng per coverslip) constructs were co-transfected and imaged 24 h later. All transfections were done using Lipofectamine 2000 (Invitrogen).

### Solutions and chemicals

The standard extracellular Ringer's solution used for electrophysiological experiments contained 130 mM NaCl, 4.5 mM KCl, 20 mM CaCl_2_, 10 mM tetraethylammoniumchloride (TEA-Cl), 10 mM D-glucose and 5 mM HEPES (pH 7.4 with NaOH). For the FRET and confocal imaging studies, the Ringer's solution was changed to one containing 2 mM CaCl_2_ and 150 mM NaCl with the other components as above. The DVF Ringer's solution contained 150 mM NaCl, 10 mM HEDTA, 1 mM EDTA, 10 mM TEA-Cl and 5 mM HEPES (pH 7.4). The internal solution contained: 135 mM Cs aspartate, 8 mM MgCl_2_, 8 mM Cs-BAPTA and 10 mM HEPES (pH 7.2 with CsOH). For the Ag^+^ block experiments, the external (divalent free) solution contained: 120 mM NaNO_3_, 10 mM EDTA and 10 mM HEPES (pH 7.4). Ag^+^ was added to this NaNO_3_-based divalent free solution at the appropriate amount calculated from the Max-Chelator software (WEBMAXC 2.10). A concentrated stock (100 mM) of AgNO_3_ in water protected from the light was prepared freshly for each experiment and diluted as needed to obtain the working concentration.

### Electrophysiology

Currents were recorded in the standard whole-cell configuration at room temperature on an Axopatch 200B amplifier (Molecular Devices) interfaced to an ITC-18 input/output board (Instrutech). Routines developed by R. S. Lewis (Stanford) on the Igor Pro software (Wavemetrics) and modified by M. Prakriya were employed for stimulation, data acquisition and analysis. Data are corrected for the liquid junction potential of the pipette solution relative to Ringer's in the bath (−10 mV). The holding potential was +30 mV. The standard voltage stimulus consisted of a 100-ms step to –100 mV followed by a 100-ms ramp from –100 to +100 mV applied at 1 s intervals. I_CRAC_ was typically activated by passive depletion of ER Ca^2+^ stores by intracellular dialysis of 8 mM BAPTA. All currents were acquired at 5 kHz and low pass filtered with a 1 kHz Bessel filter built into the amplifier. All data were corrected for leak currents collected in 100–200 μM LaCl_3_.

### Data analysis

Analysis of current amplitudes was typically performed by measuring the peak currents during the −100 mV pulse. Reversal potentials were measured from the average of several leak-subtracted sweeps (4–6) in each cell. In cases where the *I*–*V* curve asymptotically approached the *x* axis with no clear reversal (for example, in some WT Orai1 expressing cells), the reversal potential was assigned as +80 mV. Fractional blockade of current was quantified as: blockade=(1−*I*_b_/*I*_Ctrl_), where *I*_b_ is the Orai1 current in the presence of Cd^2+^ or following Ag^+^ administration, and *I*_Ctrl_ is the Orai1 current before application of the blocker.

The second order reaction rate for Cd^2+^ modification of cysteines were derived from measurements of the time constant of blockade and the extent of current inhibition. Assuming that Cd^2+^ accesses a single site from the extracellular side at a rate constant *k*_on_ and exits with a rate constant *k*_off_, and that the maximal block elicited when the cysteines are fully reacted is 100%, it follows that the time constant of blockade is given by:


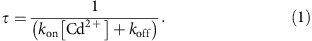


The fraction of channels that are blocked is given by:


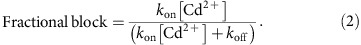


From these equations, it follows that


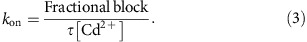


### FRET microscopy

HEK293 cells transfected with Orai1-YFP and CFP–CAD cDNA constructs (1:1 by mass) and were imaged using wide-field epifluorescence microscopy on an IX71 inverted microscope (Olympus, Center Valley, PA, USA). Cells were imaged with a × 60 oil immersion objective (UPlanApo NA 1.40), a 175 W Xenon arc lamp (Sutter, Novatao, CA, USA), and excitation and emission filter wheels (Sutter, Novato, CA, USA). At each time point, three sets of images (CFP, YFP and FRET) were captured on a cooled EM-CCD camera (Hamamatsu, Bridgewater, NJ, USA) using optical filters specific for the three images as previously described^47^. Image acquisition and analysis was performed with Slidebook software (Imaging Innovations, Denver, CO, USA). Images were captured at 3 s intervals at an exposure of 200–400 ms with 1 × 1 binning. Lamp output was attenuated to 25% by a 0.6 ND filter in the light path to minimize photobleaching. All experiments were performed at room temperature.

FRET analysis was performed using the E-FRET method described by Zal and Gascoigne^48^. The microscope-specific bleed-through constants (*a*=0.10; *b*=0.008; *c*=0.002 and *d*=0.38) were determined from cells expressing cytosolic CFP or YFP alone^47^. The apparent FRET efficiency was calculated from background-subtracted images using the formalism^48^:


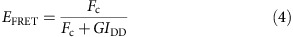


where, *F*_c_*=I*_DA_*−aI*_AA_*−dI*_DD_.

*I*_DD_, *I*_AA_ and *I*_DA_ refer to the background-subtracted CFP, YFP and FRET images, respectively. The instrument dependent *G* factor^47^ had the value 1.75±0.3. E-FRET analysis was restricted to cells with YFP/CFP ratios in the range of 2–6 to ensure that E-FRET was compared across identical acceptor to donor ratios^48^, and measurements were restricted to regions of interest (ROIs) drawn around the plasma membrane.

### Confocal microscopy

HEK293 cells expressing CFP–CAD and the various Orai1-YFP mutants were imaged on an Andor XDI Revolution spinning-disk confocal microscope equipped with a × 100 oil immersion objective. Cells were excited with 445 nm (CFP) and 515 nm (YFP) laser diodes, and the intensity of laser light was attenuated to 35% for CFP and 15% for YFP. Images were obtained at 512 × 512 pixels at an exposure of 300 ms per frame and a slice thickness of 0.8 μm. An average of five frames were used for each image in Supplementary Fig. 6. The image acquisition parameters were kept constant across all experiments. Images analysis was performed using NIH ImageJ software (NIH, Bethesda, MD, USA).

### Molecular dynamics simulations

Atomistic molecular models were constructed using the crystal structure of the *Drosophila melanogaster* Orai channel (4HKR)^10^. Missing residues of the M1-M2 loop (amino acids 181–190) and the M2–M3 loop (amino acids 220–235) were modelled *de novo* using MODELLER^49^. The C terminus was truncated at residue 329 for all chains and the N and C termini were acetylated and amidated, respectively. The protein was embedded within a hydrated dipalmitoylphosphatidylcholine (DPPC) bilayer with 150mM NaCl to obtain a cell of dimensions 120.2 × 120.2 × 117.0 Å. Pore water molecules were not modelled in any initial channel conformations. The simulation cell consisted of ∼168,000 atoms. Single point mutations were made using the NAMD tool *psfgen* to create the V174A, F171Y and F171V mutants. The CHARMM36 force field was used for protein^50^,^51^, ions and lipids^52^ along with the TIP3P water model^53^.

All simulations were conducted in the NPT ensemble (323.15 K and 1 atm) using NAMD 2.9 (ref. 54) with a 2 fs timestep. Constant pressure was ensured using the Langevin piston algorithm^55^ with an oscillation and damping timescale of 100 fs and 50 fs, respectively, with a Langevin dynamics damping coefficient of 1 ps^−1^ for temperature control. All bonds with hydrogen were constrained using the SHAKE/RATTLE algorithms^56^. Nonbonded interactions were calculated using particle mesh Ewald (PME)^57^ with a 12 Å Lennard-Jones cutoff, a switch distance at 10 Å, and pair-list distance set at 16 Å. All four systems were energy-minimized for 2,500 steps, followed by all-atom protein-restrained dynamics (2.5 ns), heavy-atom restrained dynamics (2.5 ns), and backbone-only restrained dynamics (5.0 ns). For the WT channel only, an identical model was equilibrated for ∼10 ns at each stage. A force constant of 1 kcal mol^−1^ Å^−2^ was used for all restraints. Multiple simulation repeats were initiated for each of the four systems with randomized initial velocities (10 for WT, 10 for WT with longer equilibration, 10 for V174A, 25 for F171Y and 15 for F171V). Production simulations were conducted for ∼350 ns, ∼200 ns, ∼200 ns and ∼200 ns for the WT, V174A, F171Y and F171V, mutants respectively (resulting in an aggregate simulation times of 6.9 μs, 2.2 μs, 4.3 μs and 2.9 μs respectively). Analysis was performed on all simulation frames spaced at 0.5 ns after removing the first 10 ns of data from each simulation repeat. Axial coordinates were measured with respect to the centre of mass of the pore helix Cα atoms (residues 141–174). Axial histograms of water oxygen atoms, Na^+^, and Cl^−^, were computed within a cylinder of radius of 10 Å centred at the pore centre of mass. Error bars were computed using the standard error of mean over all simulation repeats.

### Data availability

We confirm that all data supporting the findings of this study are included in the paper and its Supplementary Information files and available at request from the authors. The dOrai crystal structure (PDB code ID 4HKR) was used in the MD simulations.

## Additional information

**How to cite this article:** Yamashita, M. *et al*. STIM1 activates CRAC channels through rotation of the pore helix to open a hydrophobic gate. *Nat. Commun.* **8,** 14512 doi: 10.1038/ncomms14512 (2017).

**Publisher's note:** Springer Nature remains neutral with regard to jurisdictional claims in published maps and institutional affiliations.

## Supplementary Material

Supplementary Information Supplementary Figures, Supplementary Tables.
