# Supplementary material for: STIM1 activates CRAC channels through rotation of the pore helix to open a hydrophobic gate
Source: Nat Commun. 2017 Feb 21;8:14512. doi: 10.1038/ncomms14512 (PMC5321763; doi:10.1038/ncomms14512)
Supplement: Supplementary Information — Supplementary Figures, Supplementary Tables. [file ncomms14512-s1.pdf]

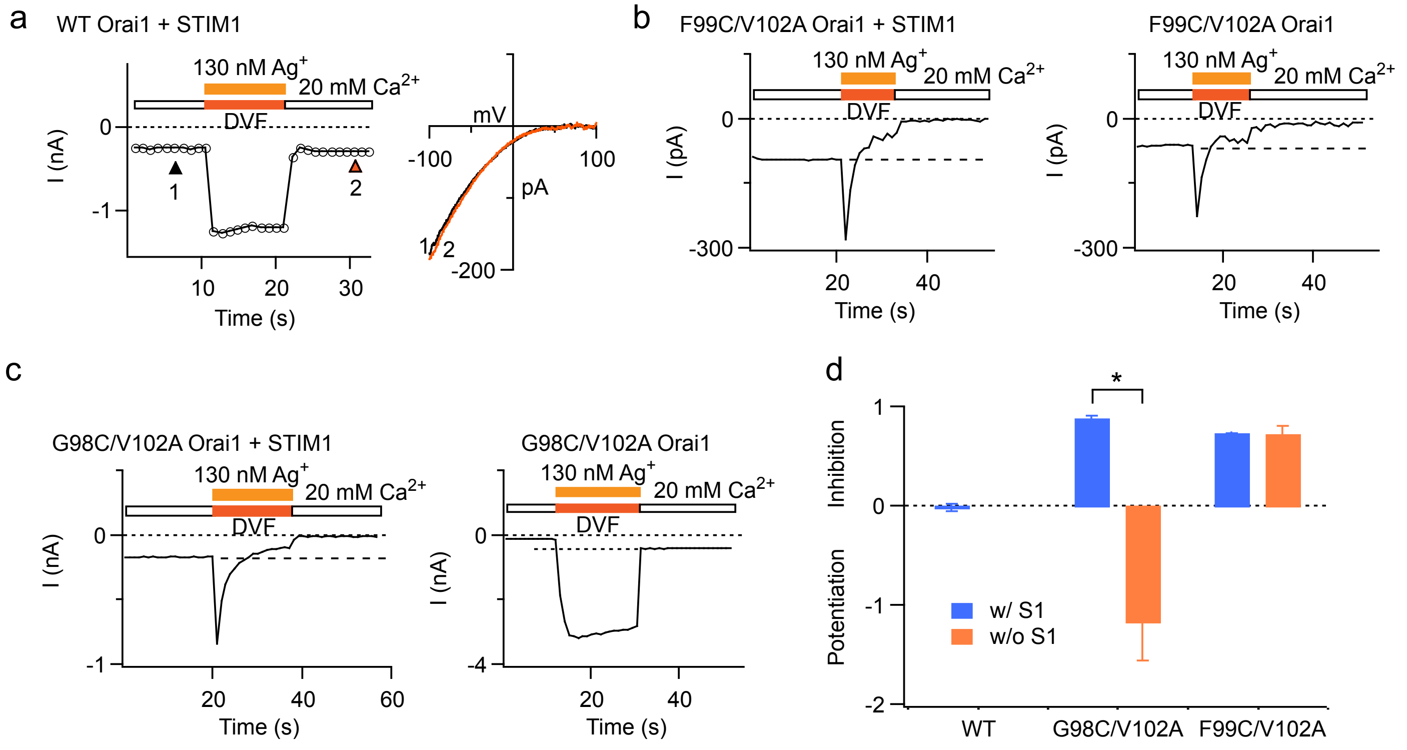

Supplementary Figure 1. Solvent accessibility of G98C and F99C revealed by Ag^+ modification of cysteine side-chains. (a) Ag^+ does not affect WT Orai1 currents. WT Orai1 was co-expressed with STIM1 in HEK293 cells. ER Ca^{2+} stores were depleted with 1 μM thapsigargin and I_{CRAC} was monitored during voltage steps to -100 mV. I-V plots collected at the time points indicated by the arrowheads are shown in the right graph. **(b)** Ag^+ (130 nM) inhibits F99C/V102A Orai1 current, both in the presence and absence of STIM1. The dotted line in each graph indicates the magnitude of the Ca^{2+} current prior to administration of Ag^+ in DVF solution. Note that administration of the DVF solution evokes a Na^+ current that is quickly blocked by Ag^+. **(c)** In the G98C/V102A mutant, Ag^+ inhibits current in the presence of STIM1 (left graph). In the absence of STIM1, however (right graph), modification by Ag^+ enhances G98C/V102A current. **(d)** Summary of inhibition (or potentiation) of Ca^{2+} currents in the indicated constructs. Inhibition (or potentiation) was measured as: $(1-I_{post}/I_{pre})$ where I_{post} is the amplitude of the current measured in 20 mM Ca^{2+}_o following administration of Ag^+ and I_{pre} is the current prior to Ag^+ application (see arrowheads in panel *a*). n=3-5 cells per condition. Values are mean ± s.e.m.

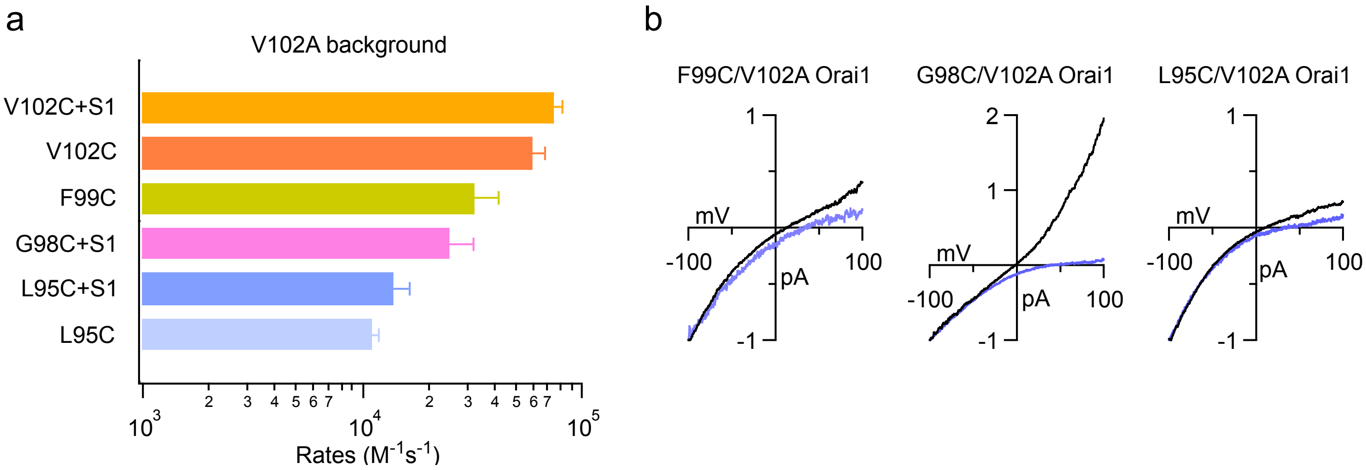

Supplementary Figure 2. **(a)** Rate constants of Cd^{2+} blockade in various Orai1 cysteine mutants. Rate constants of Cd^{2+} blockade are similar to those measured previously for TM1 pore residues[15], and comparable to the effective rate constants of the overall ion transfer process itself, ~10^5 $M^{-1}s^{-1}$ [52], suggesting that the rate of Cd^{2+} block is limited by the low unitary conductance of CRAC channels. Values are mean ± s.e.m. **(b)** Addition of STIM1 enhances the Ca^{2+} selectivity of F99C/V102A, G98C/V102A, and L95C/V102A channels. Plots of the current-voltage (I-V) relationships of mutant currents in the absence (black traces) or presence of STIM1 (blue traces). The addition of STIM1 evokes a rightward shift in the reversal potential, consistent with increased Ca^{2+} selectivity. Ramp currents were normalized to the values at -100 mV to facilitate comparison between traces.

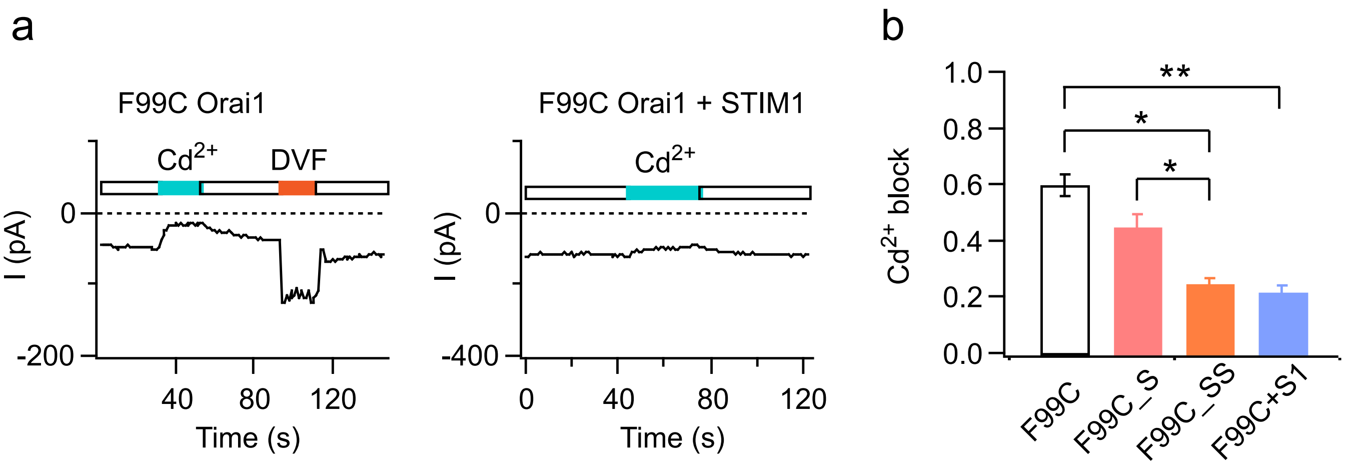

Supplementary Figure 3. STIM1 diminishes Cd^{2+} blockade of F99C Orai1 mutant channels. **(a)** The constitutive current in F99C Orai1 (in the absence of STIM1) is strongly blocked by Cd^{2+}, but block decreases in STIM1 co-expressing cells. **(b)** Summary of Cd^{2+} blockade of F99C Orai1 in the absence or presence of STIM1 co-expression and in F99C Orai1 channels tethered to either one, or two SOAR domains (Orai1-S or Orai1-SS channels). Cd^{2+} blockade dose-dependently declines with increasing SOAR (or STIM1) at the channel. *: $p<0.01$. **: <0.001, one-way ANOVA followed by unpaired T-tests. N=5-6 cells for each condition. Values are mean ± s.e.m.

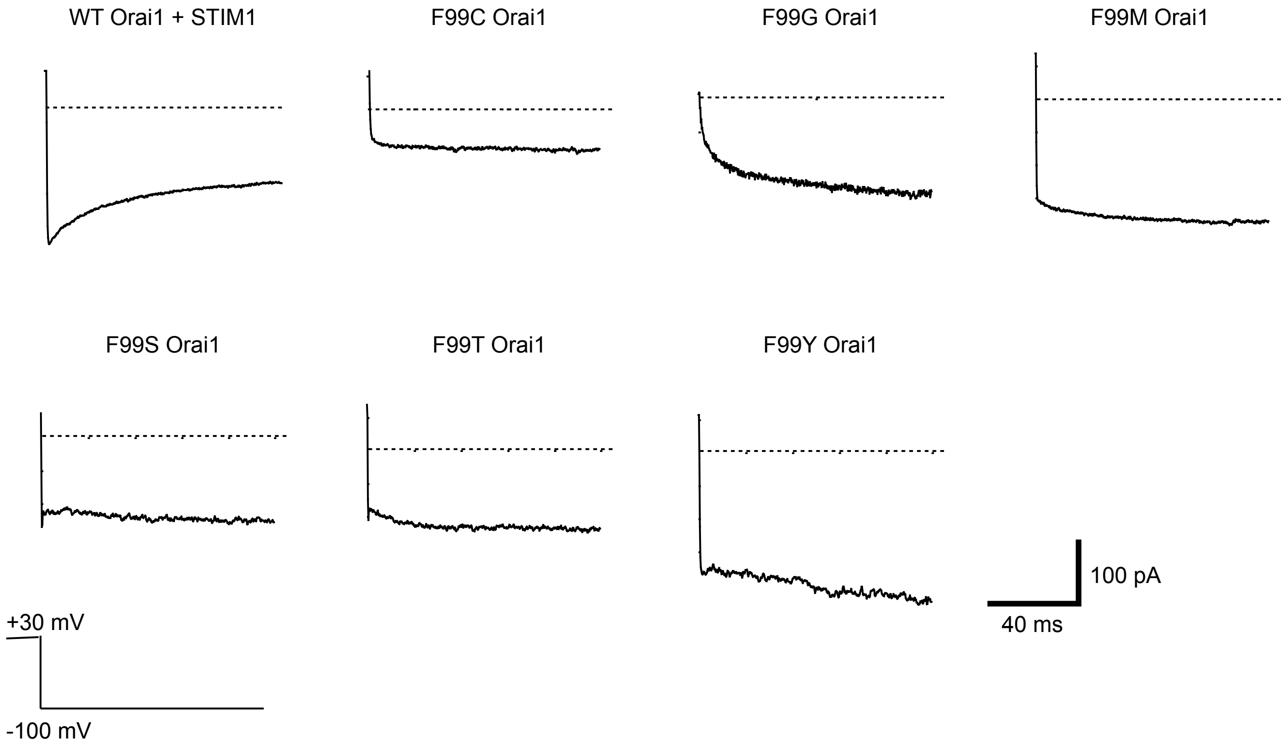

Supplementary Figure 4. F99X mutants do not exhibit Ca^{2+}-dependent fast inactivation. Currents were evoked by stepping the membrane voltage to -100 mV for 100 ms from a holding potential of +30 mV in HEK293 cells expressing either WT Orai1with STIM1 or the indicated mutant Orai1 constructs without STIM1.

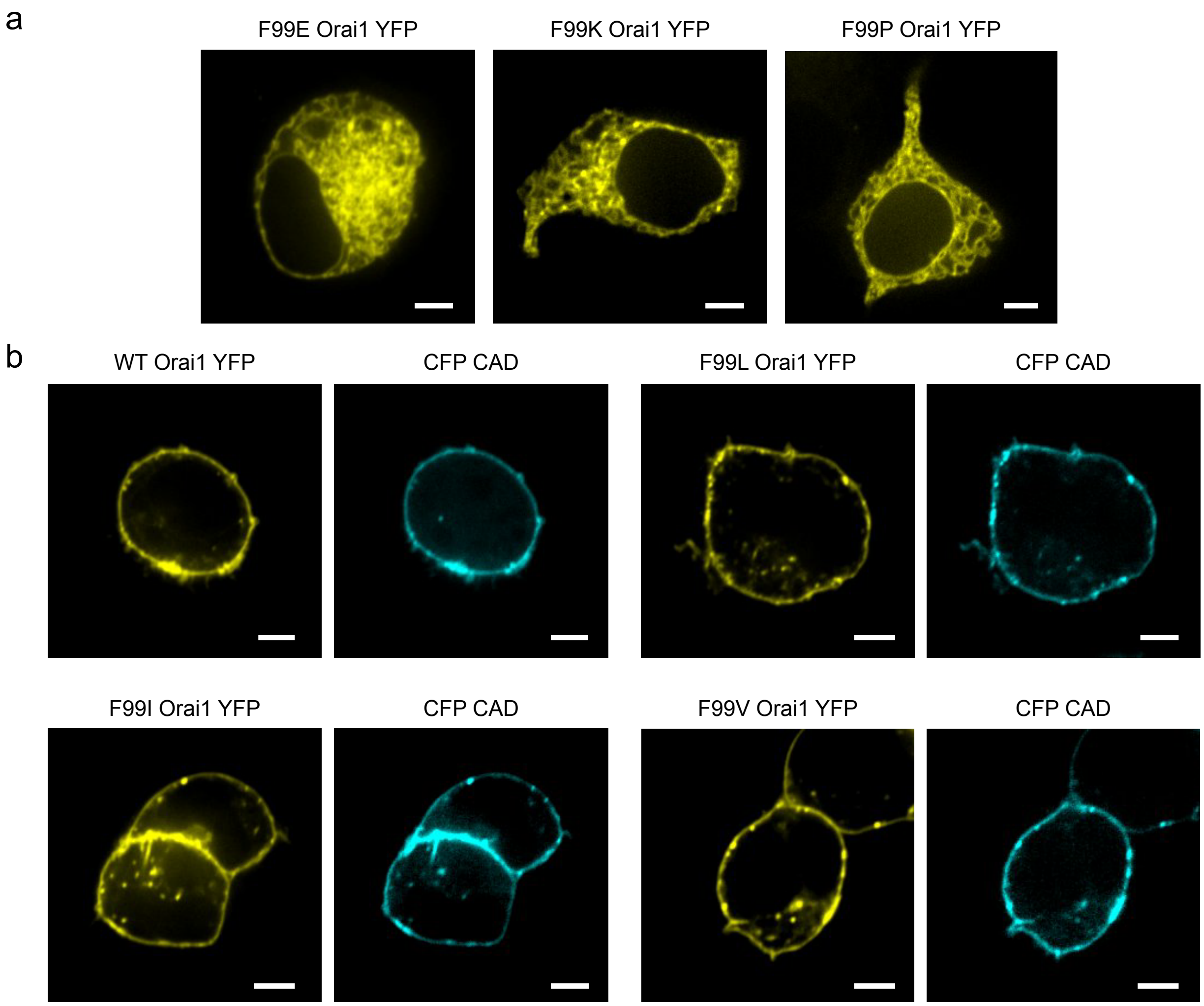

Supplementary Figure 5. (a) Confocal images showing expression of F99E, F99K, and F99P Orai1 mutants. The mutants localized primarily in an intracellular compartment, likely to be the ER. **(b)** Confocal images showing Orai1 expression of various F99X mutants and co-localization of Orai1 and CAD at the plasma membrane. The indicated Orai1-YFP constructs were expressed in HEK293 cells together with CFP-CAD. Scale bars: 5 μm.

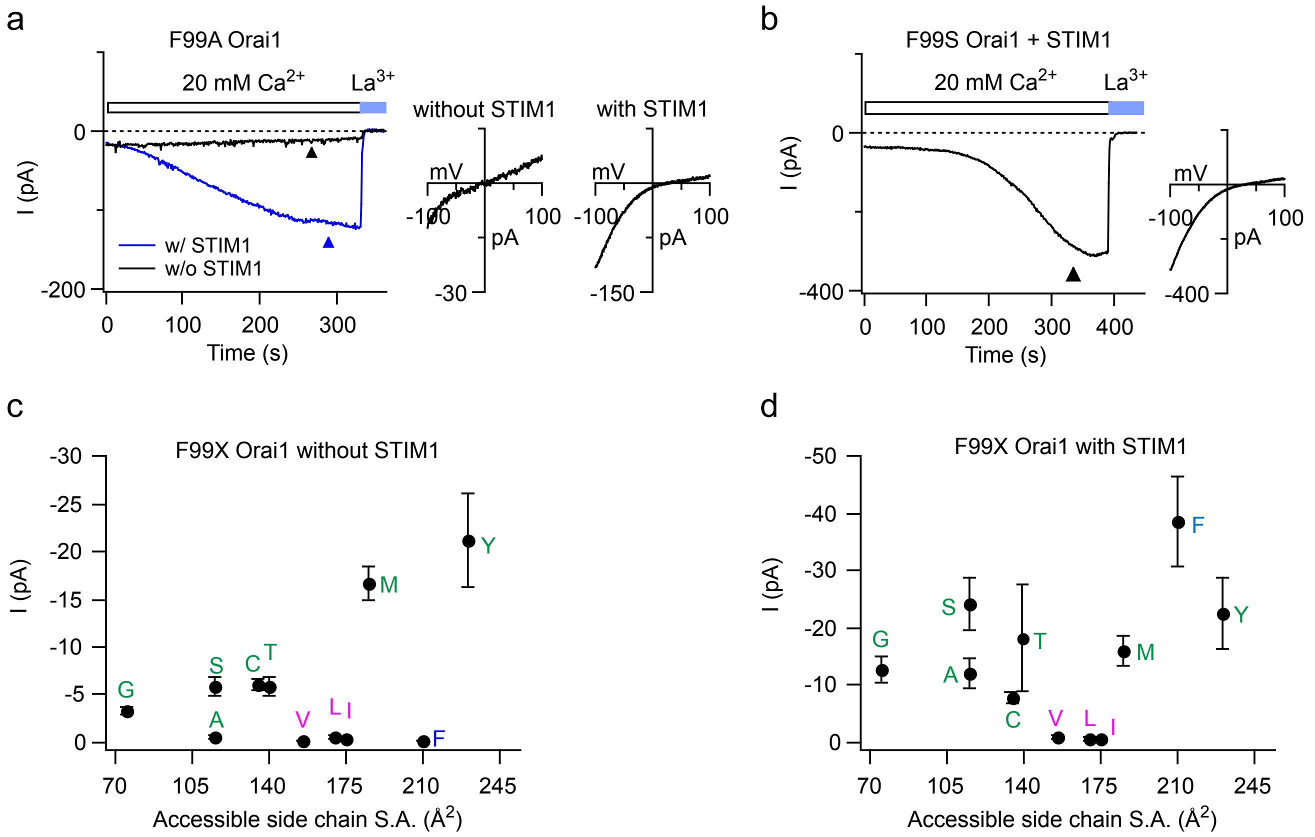

Supplementary Figure 6. Characterization of F99X mutants. (a) Store-operated induction of F99A Orai1 currents in the absence (black trace) or presence (blue trace) of STIM1. Time zero indicates the moment of whole-cell break-in. The I-V plots of the currents at the time points indicated by the arrowheads are shown in the right graphs. **(b)** Store-operated activation of F99S Orai1 in the presence of STIM1. **(c,d)** Current densities in F99X mutants plotted against the accessible side-chain surface area of the introduced amino acid in the absence *(c)* and presence *(d)* of STIM1. The native residue (Phe) is shown in blue, the constitutively conducting mutants in green, and the non-conducting mutants in magenta. N=4-6 cells for each mutant. Values are mean ± s.e.m.

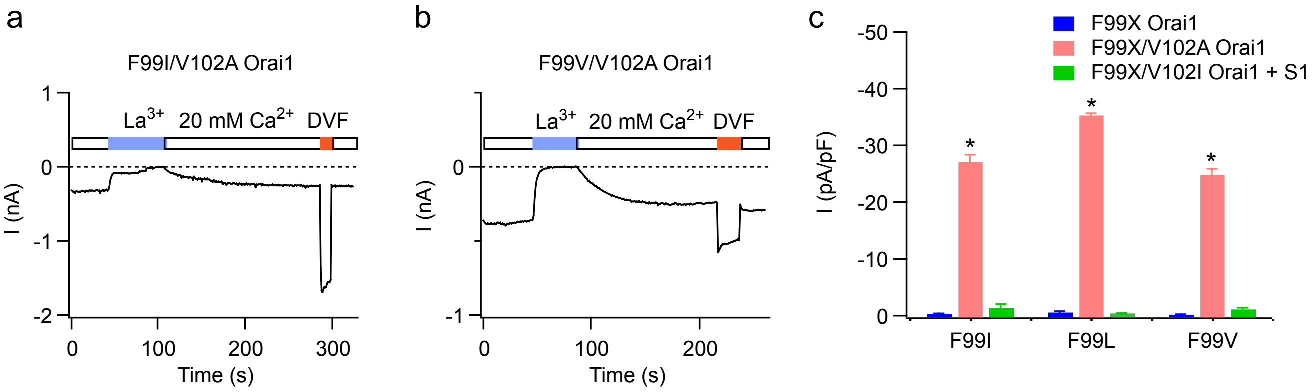

Supplementary Figure 7. Introduction of a V102A mutation restores ion conduction in the non-conducting F99I/L/V channels. (a,b) Time course of the indicated Orai1 mutants following whole-cell break-in (at time zero). **(c)** Summary of the current densities of the indicated F99X mutants in the absence or presence of the V102A mutation. In contrast to the gain-of-function effect caused by V102A on the closed F99I/L/V mutants, introduction of a V102I mutation keeps these mutants non-functional even in the presence of STIM1. N=4-5 cells per condition. *: $p < 0.001$; Student's T-test. Values are mean ± s.e.m.

Supplementary Table 1: Properties of F99X and G98X Orai1 mutants

Mutants	Reversal Potential (mV)		Current Density I (pA/pF) ± s.e.m.	
**F99X**	**w/o STIM1**	**w/ STIM1**	**w/o STIM1**	**w/ STIM1**
F99A	*	31	-0.7 ± 0.2	-12.1 ± 2.5
F99C	7	22	-5.9 ± 0.7	-6.3 ± 1.0
F99G	11	30	-3.3 ± 0.4	-12.1 ± 2.1
F99I	*	*	-0.3 ± 0.1	-0.8 ± 0.5
F99L	*	*	-0.5 ± 0.2	-0.6 ± 0.2
F99M	25	31	-16.6 ± 1.8	-16.1 ± 2.5
F99N	*	*	-0.3 ± 0.1	ND
F99S	6	24	-6.0 ± 1.0	-24.2 ± 4.5
F99T	9	17	-6.0 ± 1.0	-18.5 ± 9.0
F99V	*	*	-0.2 ± 0.1	-0.8 ± 0.4
F99W	7	*	-19.3 ± 5.1	ND
F99Y	6	37	-21.1 ± 5.0	-22.5 ± 6.3
**G98X**				
G98A	*		-0.2 ± 0.1	
G98C	*		-0.4 ± 0.2	
G98D	*		-1.4 ± 0.2	
G98E	11		-3.8 ± 1.2	
G98F	*		-0.4 ± 0.2	
G98H	-4		-3.3 ± 1.4	
G98I	*		-0.3 ± 0.2	
G98K	*		-0.5 ± 0.2	
G98L	*		-0.1 ± 0.1	
G98M	-3		-1.2 ± 0.5	
G98N	3		-5.4 ± 0.8	
G98P	-2		-6.3 ± 2.7	
G98Q	2		-16.7 ± 0.7	
G98S	8		-21.3 ± 9	
G98T	18		-13.8 ± 4.7	
G98V	*		-0.5 ± 0.3	
G98W	*		-0.2 ± 0.1	
G98Y	20		-1.3 ± 0.3	

Currents were measured in the indicated YFP-Orai1 mutants in 20 mM Ca^{2+} Ringer's solution. Reversal potentials were determined from voltage ramps (-100 to 100 mV). Current densities measured at -100 mV are shown as mean ± s.e.m; n=4–13 cells.
"*" denotes reversal potentials (or current densities) that could not be reliably measured from cells with currents < 1 pA/pF. ND: Not determined.

Supplementary Table 2: Primer sequences, V102X mutants

Orai1 Mutants		Primer sequence (5'-3')
V102A	F	GGCTTCGCCATGGCGGCAATGGTGGAG
	R	CTCCACCATTGCCGCCATGGCGAAGCC
V102C	F	CTCCGGCTTCGCCATGTGCGCAATGGTGGAGGTGC
	R	GCACCTCCACCATTGCGCACATGGCGAAGCCGGAG
L95C/V102A	F	GCCACCATGGCGCAGCAGGAGAGCAGAGC
	R	GCGAAGCCGGAGAGGCAAGCCGAGGTCCGGC
G98C/V102A	F	GGCTCTGCTCTCCTGCTTCGCCATGGC
	R	GCCATGGCGAAGCAGGAGAGCAGAGCC
F99C/V102A	F	TCTGCTCTCCGGCTGCGCCATGGCGGCAATGG
	R	CCATTGCCGCCATGGCGCAGCCGGAGAGCAGA
F99I/V102I	F	TCTGCTCTCCGGCATCGCCATGATAGCAATGGTGGAGG
	R	CCTCCACCATTGCTATCATGGCGATGCCGGAGAGCAGA
F99L/V102I	F	CCTCCACCATTGCTATCATGGCTAAGCCGGAGAGCAGAGC
	R	GCTCTGCTCTCCGGCTTAGCCATGATAGCAATGGTGGAGG
F99V/V102I	F	CCTCCACCATTGCTATCATGGCGACGCCGGAGAGCAGA
	R	TCTGCTCTCCGGCGTCGCCATGATAGCAATGGTGGAGG
WT	F	AGAGTTACTCCGAGGTGAT
	R	CTTGACCGAGTTGAGATT

F: forward primer; R: reverse primer. WT primers were used for sequencing all mutants.

Supplementary Table 3: Primer sequences, F99X mutants

Orai1 Mutants		Primer sequence (5'-3')
F99A	F	TTGCCACCATGGCGGCGCCGGAGAGCAGAG
	R	CTCTGCTCTCCGGCGCCGCCATGGTGGCAA
F99C	F	GGCTCTGCTCTCCGGCTGCGCCATGGT
	R	ACCATGGCGCAGCCGGAGAGCAGAGCC
F99D	F	TTGCCACCATGGCGTCGCCGGAGAGCAGAG
	R	CTCTGCTCTCCGGCGACGCCATGGTGGCAA
F99E	F	CATTGCCACCATGGCCTCGCCGGAGAGCAGAGC
	R	GCTCTGCTCTCCGGCGAGGCCATGGTGGCAATG
F99G	F	TTGCCACCATGGCGCCGCCGGAGAGCAGAG
	R	CTCTGCTCTCCGGCGGCGCCATGGTGGCAA
F99H	F	TTGCCACCATGGCGTGGCCGGAGAGCAGAG
	R	CTCTGCTCTCCGGCCACGCCATGGTGGCAA
F99I	F	CCACCATGGCGATGCCGGAGAGCAG
	R	CTGCTCTCCGGCATCGCCATGGTGG
F99K	F	CATTGCCACCATGGCCTTGCCGGAGAGCAGAGC
	R	GCTCTGCTCTCCGGCAAGGCCATGGTGGCAATG
F99L	F	GCCACCATGGCTAAGCCGGAGAGCAGA
	R	TCTGCTCTCCGGCTTAGCCATGGTGGC
F99M	F	CTCTGCTCTCCGGCATGGCCATGGTGGCAAT
	R	ATTGCCACCATGGCCATGCCGGAGAGCAGAG
F99N	F	TTGCCACCATGGCGTTGCCGGAGAGCAGAG
	R	CTCTGCTCTCCGGCAACGCCATGGTGGCAA
F99P	F	TTGCCACCATGGCGGGGCCGGAGAGCAGAG
	R	CTCTGCTCTCCGGCCCCGCCATGGTGGCAA
F99Q	F	CATTGCCACCATGGCCTGGCCGGAGAGCAGAGC
	R	GCTCTGCTCTCCGGCCAGGCCATGGTGGCAATG
F99R	F	TTGCCACCATGGCGCGGCCGGAGAGCAGAG
	R	CTCTGCTCTCCGGCCGCGCCATGGTGGCAA
F99S	F	TTGCCACCATGGCGCTGCCGGAGAGCAGAG
	R	CTCTGCTCTCCGGCAGCGCCATGGTGGCAA
F99T	F	TTGCCACCATGGCGGTGCCGGAGAGCAGAG
	R	CTCTGCTCTCCGGCACCGCCATGGTGGCAA
F99V	F	CCACCATGGCGACGCCGGAGAGCAG
	R	CTGCTCTCCGGCGTCGCCATGGTGG
F99W	F	CCACCATGGCCCAGCCGGAGAGCAGAGCCG
	R	CGGCTCTGCTCTCCGGCTGGGCCATGGTGG
F99Y	F	CCACCATGGCATAGCCGGAGAGCAGAGCCG
	R	CGGCTCTGCTCTCCGGCTATGCCATGGTGG

F: forward primer; R: reverse primer.

Supplementary Table 4: Primer sequences, G98X mutants

Orai1 Mutants		Primer sequence (5'-3')
G98A	F	GCTCTGCTCTCCGCCTTCGCCATGGTG
	R	CACCATGGCGAAGGCGGAGAGCAGAGC
G98C	F	GGCTCTGCTCTCCTGCTTCGCCATGGT
	R	ACCATGGCGAAGCAGGAGAGCAGAGCC
G98D	F	GCTCTGCTCTCCGACTTCGCCATGGTG
	R	CACCATGGCGAAGTCGGAGAGCAGAGC
G98E	F	GGCTCTGCTCTCCGAGTTCGCCATGGTGGC
	R	GCCACCATGGCGAACTCGGAGAGCAGAGCC
G98F	F	CCACCATGGCGAAGAAGGAGAGCAGAGCCG
	R	CGGCTCTGCTCTCCTTCTTCGCCATGGTGG
G98H	F	CCACCATGGCGAAGTGGGAGAGCAGAGCCG
	R	CGGCTCTGCTCTCCCACTTCGCCATGGTGG
G98I	F	CCACCATGGCGAAGATGGAGAGCAGAGCCG
	R	CGGCTCTGCTCTCCATCTTCGCCATGGTGG
G98K	F	CTCGGCTCTGCTCTCCAAGTTCGCCATGGTGGCAA
	R	TTGCCACCATGGCGAACTTGGAGAGCAGAGCCGAG
G98L	F	TTGCCACCATGGCGAATAGGGAGAGCAGAGCCGAG
	R	CTCGGCTCTGCTCTCCCTATTCGCCATGGTGGCAA
G98M	F	CTCGGCTCTGCTCTCCATGTTCGCCATGGTGGCAA
	R	TTGCCACCATGGCGAACATGGAGAGCAGAGCCGAG
G98N	F	CCACCATGGCGAAGTTGGAGAGCAGAGCCG
	R	CGGCTCTGCTCTCCAACTTCGCCATGGTGG
G98P	F	GGCTCTGCTCTCCCCCTTCGCCATGGTG
	R	CACCATGGCGAAGGGGGAGAGCAGAGCC
G98Q	F	TCGGCTCTGCTCTCCCAGTTCGCCATGGTGGCA
	R	TGCCACCATGGCGAACTGGGAGAGCAGAGCCGA
G98S	F	GGCTCTGCTCTCCAGCTTCGCCATGGT
	R	ACCATGGCGAAGCTGGAGAGCAGAGCC
G98T	F	CGGCTCTGCTCTCCACCTTCGCCATGGTGG
	R	CCACCATGGCGAAGGTGGAGAGCAGAGCCG
G98V	F	GCTCTGCTCTCCGTCTTCGCCATGGTG
	R	CACCATGGCGAAGACGGAGAGCAGAGC
G98W	F	GGCTCTGCTCTCCTGGTTCGCCATGGTGG
	R	CCACCATGGCGAACCAGGAGAGCAGAGCC
G98Y	F	ATTGCCACCATGGCGAAATAGGAGAGCAGAGCCGAGG
	R	CCTCGGCTCTGCTCTCCTATTTCGCCATGGTGGCAAT
G98C/F99C	F	GCCACCATGGCGCAGCAGGAGAGCAGAGC
	R	GCTCTGCTCTCCTGCTGCGCCATGGTGGC
G98C/F99C/V102A	F	CCACCATTGCCGCCATGGCGCAGCAGGAGAGCAGAG
	R	CTCTGCTCTCCTGCTGCGCCATGGCGGCAATGGTGG

F: forward primer; R: reverse primer.
